# Multilocus Sequence Typing for Molecular Epidemiology of *Stenotrophomonas maltophilia *Clinical and Environmental Isolates from a Tertiary Hospital in West of Iran

**DOI:** 10.52547/ibj.26.2.142

**Published:** 2022-01-26

**Authors:** Sinasadat Emami, Jamileh Nowroozi, Ramin Abiri, Parviz Mohajeri

**Affiliations:** 1 Microbiology Department of Islamic Azad University, North Tehran Branch, Iran;; 2 epartment of Microbiology, Kermanshah University of Medical Science, Kermanshah, Iran

**Keywords:** Environment, Multilocus sequence typing, Stenotrophomonas* maltophilia*

## Abstract

**Background:**

*Stenotrophomonas maltophilia* is an opportunistic bacterium, contributing to different hospital-acquired infections and can be acquired from different hospital setting sources. Epidemiological study of *S. maltophilia *in the hospital also demonstrates the intrahospital distribution of certain strains of bacteria in healthcare facilities. The aim of the current study was to identify the molecular epidemiology of *S. maltophilia* isolates from clinical and environmental sources within a hospital.

**Methods:**

A total of 400 samples (clinical and environmental) were collected from the different settings of hospital. Following the standard biochemical testing and 23S rRNA genotyping, the molecular typing of *S. maltophilia* isolates was determined using the MLST technique. Also, the frequencies of *zot* and *entF* virulence genes among *S. maltophilia* isolates were examined by PCR technique.

**Results:**

Based on the biochemical testes and PCRs targeting 23S rRNA gene, 22 *S. maltophilia* isolates were identified. The MLST analysis demonstrated that these isolates were assigned to 14 ST, and 6 out of 14 STs were common among clinical and environmental samples. All 22 isolates were identified in the PubMLST database. The PCR screening demonstrated that none of 22 *S. maltophilia* isolates had *zot* virulence gene, while the *entF* gene with the 59% frequency was observed in 13 out of 22 isolates. Among these 13 isolates, 6 STs were common in clinical and environmental isolates.

**Conclusion:**

Our study showed the clonal relatedness between clinical and environmental sources of the *S. maltophilia* isolates in a hospital. Further studies are required to understand the epidemic situation of this pathogen in the clinic and the environment.

## INTRODUCTION

*Stenotrophomonas maltophilia*, as an anaerobic, non-fermentative and Gram-negative bacteria, is a ubiquitous species of the gamma subdivision of Proteobacteria^[^^1^^,^^2^^]^. This environmental bacterium is found in foods, animals, plant rhizospheres, and aqueous environments, as well as in contaminated medical care fluids and water sources^[^^1^^,^^3^^]^. *S. maltophilia*, as an opportunistic pathogen, has been isolated from various water-based sources either inside or outside of the hospital environment or clinical settings, with a broad geographical distribution^[^^4^^,^^5^^]^ Despite the limited pathogenicity of this emerging bacterium^[^^4^^,^^6^^]^, it is responsible for different community-acquired and hospital-related diseases, especially in immunocompromised individuals, with a mortality rate of 37.5%^[^^4^^,^^7^^]^. The prevalence of the bacterium among tracheal samples in Iran is estimated to be about 4.5%. 

*S. maltophilia* can infect different organs and tissues^[^^8^^]^ and is associated with numerous clinical manifestations such as bacteremia, pneumonia, arthritis, sepsis, meningitis, endocarditis, endo-phthalmitis, urinary and respiratory tract infections^[^^9^^,^^10^^]^. Patients hospitalized at intensive care units are more vulnerable to the *S. maltophilia *infection than healthy people^[^^11^^-^^13^^]^. Based on a report from Iran, the high frequency of bacteria was found in bloodstream infections (88.6%). However, a lower rate (11.4%) of the bacteria was detected in general medicine wards^[^^14^^]^. Hence, *S*. *maltophilia *is not a prevalent pathogen, and its imputed virulence factors, including biofilm formation, motility, adhesion capacity, hydrophobicity, and synthesis of extracellular enzymes, are responsible for the inflammatory response^[^^6^^,^^15^^]^. Recently, increment of *S. maltophilia* isolation from different hospitals has contributed to uncontrolled and even exploited administration of antibiotics^[^^4^^,^^15^^]^. Since this bacterium has a role in the high level of antibiotic resistance^[^^10^^,^^16^^,^^17^^]^, there is an increasing demand for new treatment options^[^^10^^,^^18^^]^.

High genetic diversity strains have been identified among *S. maltophilia* using a variety of biomolecular techniques. To discover the relationship of clinical isolates with environmental sources, several genotypic profiling methods have been employed. A number of these methods include whole genome sequencing analyses, amplified fragment length polymorphism fingerprinting, PCR-restriction fragment length polymorphism, the gyrase B gene analysis, and PCR-based fingerprinting methods, such as BOX-A1R-based repetitive extragenic palindromic-PCR, Rep-PCR, enterobacterial repetitive intergenic consensus PCR, PFGE analysis of XbaI genomic digests, and MLST^[^^4^^,^^19^^]^. The MLST is administered to find the source of infections along with the distribution patterns of pathogens isolated from hospitalized patients. Therefore, it can provide reputable data from epidemiological distribution of the bacteria. Moreover, due to the availability of MLST data in public databases, the obtained results can be compared with other laboratories^[^^20^^]^. The aim of the current study was to identify *S. maltophilia* isolated from clinical and environmental sources within a hospital using MLST technique in order to analyze the molecular and epidemiological characteristics of this bacterium and examine the clonal relatedness between clinical and environmental specimens of the *S. maltophilia* isolates collected from hospital.

## MATERIALS AND METHODS

**Sample collection**

*S. maltophilia* isolates were collected from different clinical and environmental settings in Imam Reza hospital (a 515-bed university-affiliated tertiary hospital in Kermanshah, West of Iran) over a 12-month duration from May 2019 to May 2020. The clinical specimens, including sputum, blood, and urine, were obtained from patients hospitalized in the hospital. Environmental samples were collected from equipment, surfaces, and solutions by rubbing sterile swabs on the 10-cm^2^ surface of each selected area^[^^21^^]^.

**Biochemical identification of** ***S. maltophilia*** **isolates**

Clinical isolates were subcultured on Blood and MacConkey agars (Merck, Germany). Environmental isolates were cultured on blood agar plates and then were incubated at 37 °C for 24 hours. The standard biochemical testing, such as oxidase and catalase tests, was employed for the laboratory identification of the isolates, along with deoxyribonuclease test, triple sugar iron agar, and Sulfide Indole Motility (Merck). *S. maltophilia* ATCC 13637 was used as the control strain. All the isolates were stored in a Luria Bertani broth containing 20% glycerol at -70 °C^[^^22^^]^. 

**Molecular testing of** ***S. maltophilia*** **isolates**

***DNA extraction***

The* S. maltophilia *isolates were cultured with aeration in Luria Bertani at 37 °C overnight. Next, total DNA extraction was performed using the high pure PCR Template Preparation Kit (Roche, Germany) according to the manufacturer’s protocol. The concentrations of all DNA samples were determined by NanoDrop ND-100 (NanoDrop Technologies, USA). 

***PCR analysis***

The molecular identification of the* S. maltophilia* strain was confirmed by the detection of 23sr RNA using a common PCR method. Primers, including 23srRNA forward: 5’*CTGGATTGGTTCTAGGAAAA CGC*3’ and 23srRNA reverse: 5’*ACGCAGTCACTCCT TGCG*3’, were applied in PCR reaction using a PCR kit (QIAGEN, Hilden, Germany) with the following thermal cycles: 94 °C (5 min) followed by 36 cycles of 94 °C (45 s), 58 °C (45 s), 72 °C (45 s), and final extension step at 72 °C (5 min). The PCR products were separated and visualized on 1% agarose gel. Thereafter, samples showing the related fragments were verified by sequencing (Bioneer, Korea). 

**Molecular typing of** ***S. maltophilia*** **isolates by MLST technique**

The MLST technique was performed for the molecular typing of *S. maltophilia* isolates as described earlier^[^^23^^]^.

**Selection of housekeeping gene**

Seven pair primers targeting the conserved regions of seven housekeeping genes of *S. maltophilia* were selected from MLST website (http://pubmlst.org/ smaltophilia/). The genes included H (+)-transporting two-sector *atpD*, *gapA*, *guaA*, *mutM*, *nuoD*, *ppsA*, and *recA*. The PCR amplification of target fragments was performed as follows: initial denaturation at 94 °C (5 min) followed by 36 cycles of denaturation at 94 °C (45 s), annealing at 58.5 °C (45 s) for *atpD*, 57 °C for *gap*A, 57.5 °C for *mutM* gene, 56 °C for *mutM* and *nuoD* genes, 60 °C for *ppsA* gene, and 59.5 °C for *recA* gene, and extension step at 72 °C (45 s), with a final extension step at 72 °C (5 min). The PCR products were analyzed on agarose gel and were then sequenced by Bioneer Company (South Korea).

**Data analysis**

The obtained sequences were submitted to PubMLST database (https://pubmlst.org/) to determine the allele number and specific ST^[^^24^^]^. All unique sequences were assigned with an allele number, and each unique combination of seven alleles in each isolate was assigned a ST^[^^25^^]^.

**Phylogenetic analysis**

The statistical analysis of allele profiles and sequence data was conducted using START 2.0 software to calculate guanine-cytosine content, frequencies of alleles, number of variable sites, and the dN/dS ratio (nonsynonymous per synonymous substitutions)^[^^24^^]^. Phylogenetic tree was constructed by the neighbor-joining method in MEGA v.7 software (www.megasoftware.net). The dendrogram was constructed based on the UPGMA. 

**Frequency of** ***zot*** **and** ***entF*** **virulence genes among** ***S. maltophilia*** **isolates**

The frequency of *zot* and *entF* genes among *S. maltophilia* isolates was examined by PCR with the following primer sets: *zot* forward: 5’*GAATCGTGCTT TGATCTGC* 3’ and *zot* reverse: 5’*AGAATACCGAGGT GTACGA* 3’ primers and were used for the amplification of *zot* gene with following program: 94 °C (5 min) and 35 cycles of 94 °C (45 s), 60 °C (45 s), 72 °C (45 s), and final extension step at 72 °C (5 min). The primers *entF* forward: 5’ *GATTACGCAAC GCTGGAAG* 3’ and *entF *reverse: 5’ *CACGATGTTC GATGACCACG* 3’ were used for the PCR amplification of *entF* gene similar to *zot*except for the annealing temperature, which changed to 56 °C.

## RESULTS

**Bacterial isolates**

**Fig. 1:**
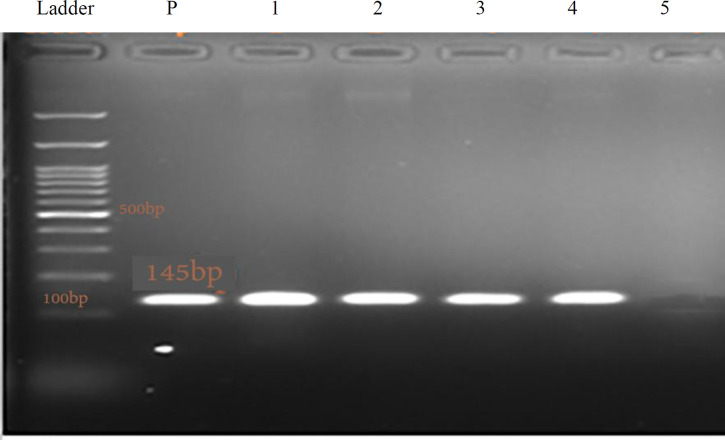
The PCR products of 23S rRNA gene electrophoresed on 1% agarose gel. The related fragment (145 bp) was observed and verified by sequencing. P, positive control; lanes 1-4, experimental samples; lane 5, negative control; ladder (100 bp)

During one year of the study, 400 samples were collected from different sites of the hospital. Twenty-two *S. maltophilia* isolates were identified based on the biochemical tests and confirmed by PCR targeting 23S rRNA gene (Fig. 1). These isolates were obtained from clinical specimens and environmental sites; 10 out of 22 isolates were identified from clinical specimens (sputum, blood, and urine). The rest was obtained from dry (catheter, dialysis machine, manometer, ventilator, thermometer, stethoscope, suction tubes, and patient beds) and moist (bathtub, ice maker, tap, water reservoir, refrigerators, showerheads, sink drains, iodine, and detergents) sites. Among the clinical specimens, *S. maltophilia* isolates obtaining from sputum samples had the highest frequency, while the frequency of *S. maltophilia* isolated from different environmental samples was almost equal. 

**MLST analysis**

**Fig. 2:**
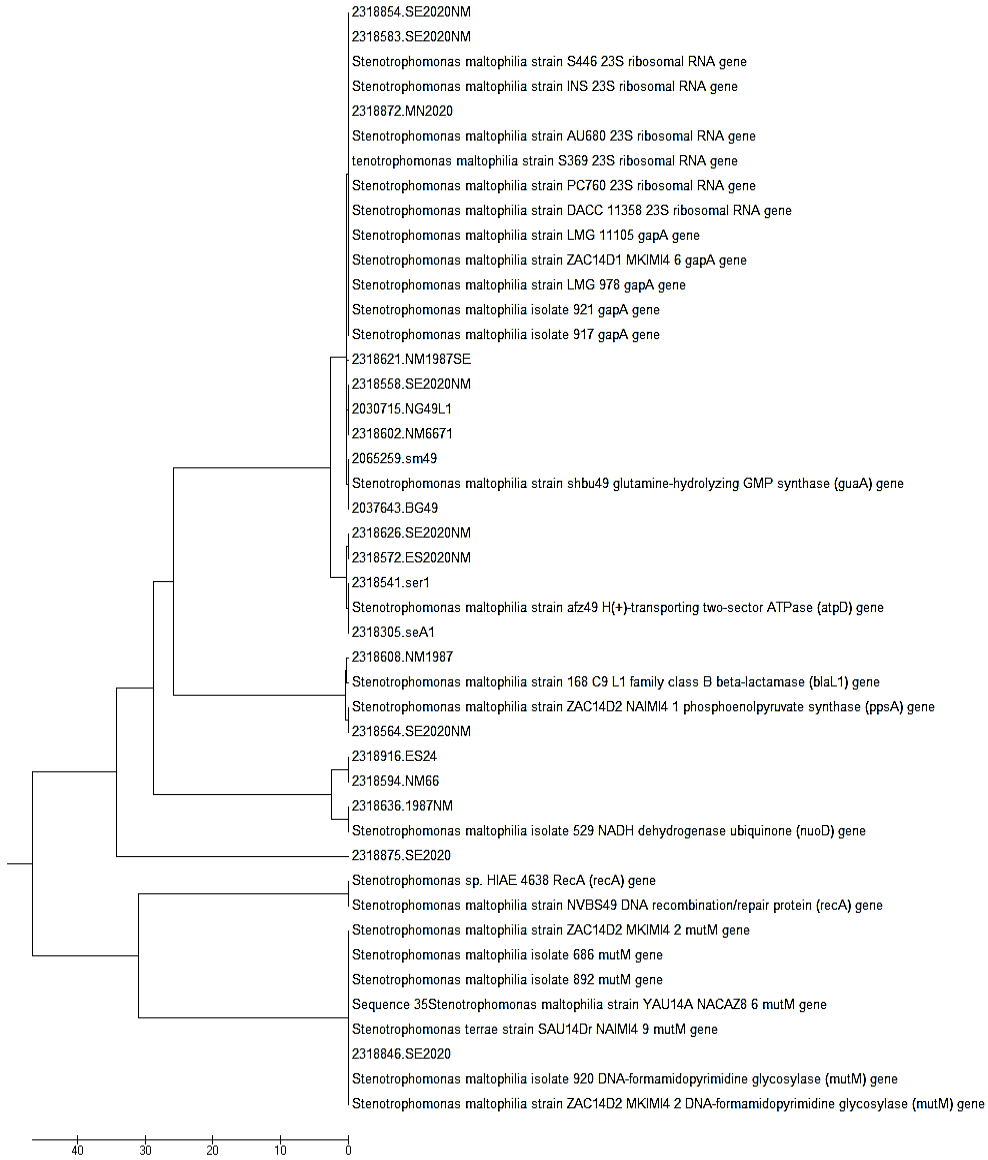
Phylogenetic trees based on the concatenated data for all seven housekeeping genes of the 22 *S. maltophilia* isolates

**Fig. 3:**
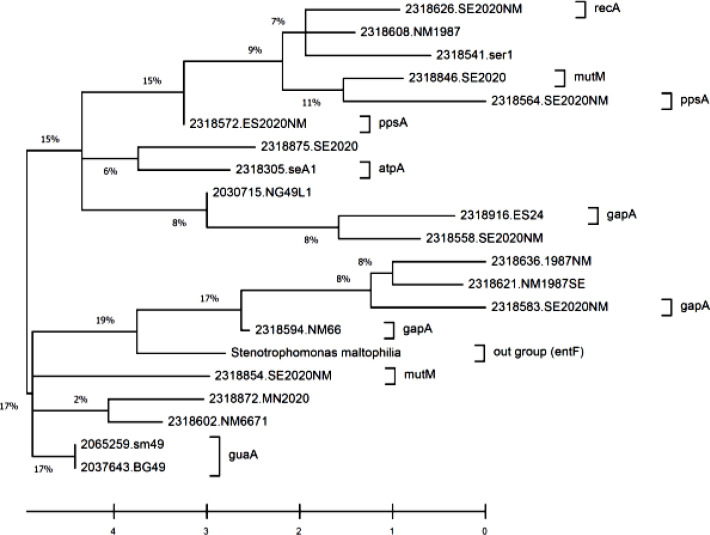
Dendrogram using UPGMA method applied by the MEGA v.7 program to show similarity between 22 isolates and other *S. maltophilia* strains obtaining from NCBI database

**Supplementary Fig. 1:**
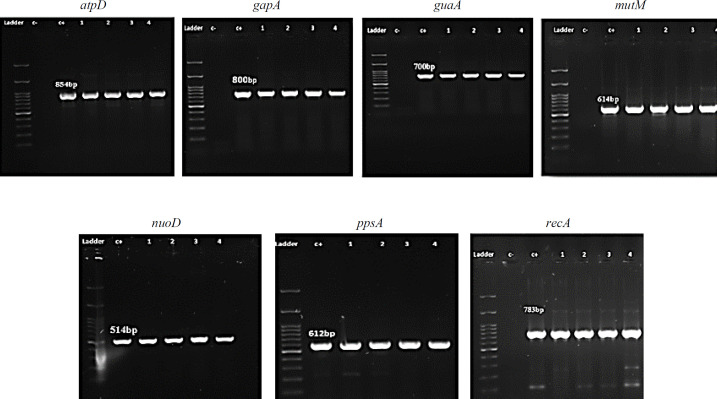
The PCR products of seven housekeeping genes amplification electrophoresed on 1% agarose gel. The related fragments of *atp*D (854 bp), *gap*A (800), *gua*A (700 bp), *mut*M (614 bp), *nuoD* (514), *ppsA* (612 bp), and *recA* (738 bp) were verified by sequencing. C+, positive control; C-, negative control; lanes 1-4: experimental samples; ladder (100 bp)

Amplification of seven housekeeping genes was conducted by PCR, and the results were observed on 1% agarose gel (supplementary Fig. 1). The fragments of these selected genes, ranged from 514 bp (*nuoD*) to 800 bp (*gapA*), were successfully sequenced and analyzed by MLST for all the obtained isolates. These sequences were submitted to PubMLST database for the determination of the allele number and ST. According to different alleles, 22 isolates were assigned to 14 STs, including ST 14, ST 15, ST 34/194, ST 84/482, ST 85/99, ST 92, ST 143, ST 186, ST 186/252, ST 178, ST 196, ST 300, ST 451/461, and ST 477. All the 22 isolates consisted of existing types in the PubMLST database. The details of ST and allele numbers are shown in Table 1. The MLST results of 22 *S. maltophilia* isolates were demonstrated in phylogenetic tree using a neighbor-joining tree analysis for the concatenated data in all seven housekeeping genes of the 22 isolates (Fig. 2). UPGMA dendrogram demonstrated the similarity between these 22 isolates and other *S. maltophilia* strains obtaining from NCBI database (Fig. 3). 

**Determination of** ***zot*** **and** ***entF*** **gene frequency in** ***S. maltophilia*** **isolates**

**Fig. 4:**
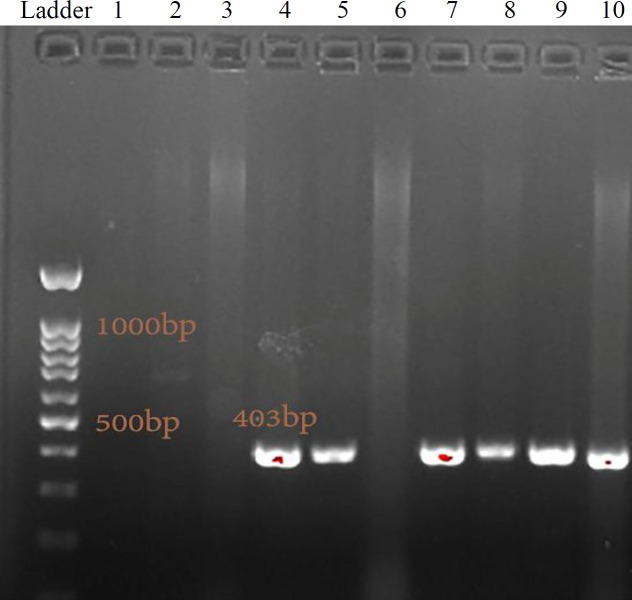
The PCR products of *entF* gene. The related fragment (403 bp) observed on 1% electrophoresed agarose gel. Lane 1, negative control; lane 10, positive control; lanes 2-9, experimental samples; lanes 4, 5, 7, 8, 9, experimental samples containing *entF* gene; ladder (100 bp)

The PCR method was employed to determine the frequency of *zot* and *entF* virulence genes among 22 isolates of *S. maltophilia. *None of 22 isolates were positive for the presence of *zot* gene, indicating the absence of *zot* virulence factor. However, 13 out of 22 isolates were positive for the *entF* gene, representing the frequency of 59% for this virulence factor among 22* S. maltophilia* isolates (Fig. 4). These isolates were allocated to ST 15, ST 92, ST 178, ST 186, ST 196, ST 85/99, ST 186/252, ST 84/482, ST 300, ST 451/461, and ST 477. It should be noted that the *entF *gene was observed only in sputum specimen. The detailed information on the determination of *zot* and *entF* genes frequency in *S. maltophilia* isolates is available in Table 2.

**Table 1:** Allele numbers and STs of* S. maltophilia* isolates obtained by MLST

**Sampling site**		**Allelic profile**								**ST number**
		***recA***	***guaA***	***gapA***	***nuoD***	***ppsA***	***MutM***	***atpD***		
Clinical specimen		66	138	96	7	38	46	5		196
		62	82	94	28	4	3	5		84/482
		141	223	66	62	68	45	2		300
		16	284	70	18	15	33	28		85/99
		80	265	124	72	111	112	13		477
		80	49	124	92	144	83	13		186/252
		6	18	1	1	4	6	1		178
		6	23	23	26	5	16	17		14
		16	166	8	4	167	33	116		451/461
		26	10	10	23	1	14	10		34/194
										
Dry environment		5	223	66	62	68	45	2		300
		80	265	124	72	111	112	91		477
		1	82	1	1	1	3	1		92
		16	166	8	4	167	33	116		451/461
		10	21	29	32	32	21	10		15
		6	138	104	7	80	46	3		196
										
Moist environment		1	82	1	1	1	3	1		92
		106	18	1	1	4	6	1		178
		80	97	124	140	110	74	13		143
		5	223	66	62	68	45	2		300
		80	290	99	92	111	83	13		186
		1	82	1	1	23	3	10		92

## DISCUSSION

In the past decades, the prevalence of *S*. *maltophilia *has increased worldwide due to the misuse of antibiotics, especially in hospitals^[^^8^^,^^15^^,^^18^^]^. This nosocomial bacterium form a biofilm to be resistant against antibiotics, resulting in a high prevalence of antibiotic resistant strains^[^^23^^]^. The clinical importance of *S.* *maltophilia*, as a mere colonizer or infectious agent, often remains unresolved^[^^24^^]^. Hence, the origin and the transmission way of this pathogen between patients are necessary to be elucidated.

Healthcare-associated infections with *S. maltophilia *have been originated from different hospital setting sources, such as hemodialysis water and dialysis machine^[^^26^^]^, endoscopes^[^^27^^]^, contact lens solutions^[^^28^^]^, and contaminated disinfectants^[^^29^^]^, as well as handwashing soap^[^^30^^]^, sinkholes^[^^31^^]^, sink drains, and showers^[^^32^^]^. The transmission of pathogen could be performed by direct contact with these infected clinical settings and/or by the healthcare personnel’s hands^[^^21^^]^. The study on the epidemiology of *S. maltophilia *in hospital can demonstrate the intrahospital dissemination of distinct isolates of this pathogen in the clinic and environment. This study is helpful to control the increasing frequency of pathogen among hospitalized patients. 

**Table 2:** Distribution of *zot* and *entF *virulence genes among STs of *S. maltophilia*

**Source**		**Virulence genes**			**ST number**
		***zot***	***entF***		
Clinical specimen		-	+		ST 196, ST 84/482, ST 300, ST 85/99, ST 186/252, ST 178, ST 451/461
					
Dry environment		-	+		ST 477, ST 451/461, ST 15, ST 196
					
Moist environment		-	+		ST 92, ST 186

Genotyping methods have been used successfully in the molecular epidemiology of *S. maltophilia *and have revealed the high genodiversity of this species^[^^25^^]^. Since MLST is considered as one of the best methods to study molecular epidemiology and population structure of bacteria^[^^25^^,^^33^^]^, it can be used to investigate the epidemiology of *S. maltophilia* in healthcare unites such as hospitals. In a study conducted by Bostanghadiri *et al.*^[^^4^^]^, the genotypic characterization of 164 *S. maltophilia* isolates, collected from hospitalized patients in various regions of Iran, were determined by MLST and Rep-PCR methods. For the evaluation of genetic diversity, all 164 *S. maltophilia* isolates were divided into 16 common types and 114 single types, using Rep-PCR fingerprinting. For the first time in Iran, they showed that five TMP-SMX-resistant *S. maltophilia* isolates belonged to two different STs, including ST139 and ST259, using MLST analysis. ST259 with allelic profile (26,14,140,103,3,8,11) has not previously been reported. Their study also demonstrated the diversity among the isolates, suggesting the increment of antibiotic resistance and alternation of biofilm genes in clinical *S. maltophilia *isolates in Iran. However, TMP-SMX is still an effective antibiotic against *S. maltophilia*^[^^4^^]^. 

In the current study, we used MLST with seven housekeeping genes on 22 *S. maltophilia *isolates obtained from patients and clinical settings of a tertiary hospital in the west of Iran (Kermanshah), to analyze the molecular epidemiology as well as clonal relatedness between clinic and environment sources of the *S. maltophilia* isolates. Among 22 isolates, 10 were identified from sputum, blood, and urine as clinical specimens, and the rest was obtained from dry and moist sites of the hospital. Among clinical specimens, 80% of *S. maltophilia* isolates were obtained from sputum samples. Moreover, the frequency of *S. maltophilia* isolates collected from dry and moist sites was almost equal. Using MLST analysis, we showed that these 22 isolates were assigned to 14 STs, which all types existed in the PubMLST database. As shown in Table 2, the ST300 was observed in clinical specimens and also in samples obtaining from dry and moist sites of the hospital. The ST196, ST477, and ST451/461 were obtained from both clinical specimens and dry sites, while ST178 was obtained from clinical specimens and moist sites. In addition, ST92 observed in both dry and moist sites. We also found that 6 out of 14 STs were common among clinical specimens and samples obtained from dry sites and moist sites of the hospital. It can be concluded that there is a clonal relatedness between the clinic and environment sources of the *S. maltophilia* isolates obtained from the hospital. 

Recently, a study conducted in Iran^[^^34^^]^ demonstrated the clonal relatedness between environmental and clinical *S. maltophilia *isolates using PFGE method, indicating a wide range of genetic diversity of *S. maltophilia *strains among the clinical and environmental sources. In that study, a total of 150 *S. maltophilia *isolates from patients and 1108 environmental samples were collected from three hospitals in Tehran (capital of Iran). At first, 150 clinical and 18 environmental isolates were confirmed using phenotypic tests, then the species were confirmed by PCR of the *23S rRNA *gene. PFGE analysis displayed 24 common pulsotypes and 32 single pulsotypes. Only a small cluster was common among the clinic and environment within a hospital, indicating the existence of a common source for *S. maltophilia* to disseminate between different wards. Therefore, that study demonstrated the intra-hospital dissemination of certain isolates of *S. maltophilia* among the clinic and environment^[^^34^^]^. 

Despite the growing significance of *S. maltophilia* infections, little is known about its pathogenicity and virulence factors^[^^35^^-^^37^^]^. Studies have indicated that *S. maltophilia* strains possess traits that link them to virulence in other bacteria^[^^8^^,^^38^^,^^39^^]^. The genomes of clinical and environmental *S. maltophilia* isolates encode three homologues proteins, EntA, EntC, and EntF^[^^38^^]^. These proteins are involved in the biosynthesis of enterobactin as a catecholate siderophore that is made by enteric bacteria (e.g. *E. coli*) and Streptomyces sp.^[^^38^^,^^40^^,^^41^^]^. The pathogenic role of siderophores has been shown in many bacteria^[^^38^^,^^42^^-^^45^^]^. Moreover, there is evidence that the *S. maltophilia* siderophore is likely important in pathogenesis^[^^22^^,^^38^^,^^46^^-^^50^^]^. Toxin-related factor has been studied in *S. maltophilia *strains as a virulence factor, and a phage-encoded zonula occludens-like toxin has been considered in this pathogen^[[^^35^^-^^37^^]^. In cholera toxin defective strains of *V. cholera*, a second enterotoxin, *zot* (zonula occludens toxin), causes diarrhea in affected patients^[^^51^^]^. Interestingly, the occurrence of a zot-like gene has been reported in *S. maltophilia* strains of clinical origin by Hagemann *et al.*^[^^36^^]^ in 2006. In the current study, we performed PCR screenings to check the distribution of the *zot* and *entF* genes as virulence factors among 22 *S. maltophilia* isolates. The results demonstrated none of 22 *S. maltophilia* isolates have *zot* virulence gene, encoding zonula occludens-like enterotoxin. However, the *entF* gene with 59% frequency was observed in 13 out of 22 isolates. Significantly all six common STs, including ST300, ST196, ST477, ST451/461, ST178, and ST92, among clinical and environmental isolates from hospital represented the *entF* gene (Fig. 4 and Table 1).

In summary, this study investigated the molecular epidemiology of *S. maltophilia* isolates from environmental and clinical sources within a hospital using the MLST technique. PCR screenings was also employed to check the distribution of the *zot* and *entF* genes, as virulence factors, among *S. maltophilia* isolates. Our findings revealed a clonal relatedness between environmental and clinical specimens of the *S. maltophilia* isolates from hospital, which may be helpful in providing necessary groundwork for the prevention and treatment of *S. maltophilia* infections.

## DECLARATIONS

### ETHICAL STATEMENT

The above-mentioned sampling was in accordance with Helsinki Declaration of 2013 and approved by the Ethics Committee of Imam Reza Hospital, Kermanshah, Iran. All participants signed informed consent forms. 

### DATA AVAILABILITY STATEMENT

Data supporting this article are included within the article and supplementary file.

### AUTHOR CONTRIBUTION

SSE contributed to all experimental work, data and statistical analysis, and interpretation of data. JN contributed to conception and design. For overall supervision, RA drafted the manuscript, and PM conducted the MLST analysis and microbial evaluation. All authors have read and approved the final manuscript

### CONFLICT OF INTEREST

None declared.

### FUNDING/SUPPORT

This study has received no funding or support.
